# CJCheck Stage 1: development and testing of a checklist for reporting community juries – Delphi process and analysis of studies published in 1996–2015

**DOI:** 10.1111/hex.12493

**Published:** 2016-10-05

**Authors:** Rae Thomas, Rebecca Sims, Chris Degeling, Jackie M. Street, Stacy M. Carter, Lucie Rychetnik, Jennifer A. Whitty, Andrew Wilson, Paul Ward, Paul Glasziou

**Affiliations:** ^1^ Centre for Research in Evidence‐Based Practice (CREBP) Faculty of Health Sciences and Medicine Bond University Gold Coast Qld Australia; ^2^ Centre for Values, Ethics and the Law in Medicine University of Sydney Sydney NSW Australia; ^3^ School of Public Health The University of Adelaide Adelaide SA Australia; ^4^ School of Medicine University of Notre Dame Sydney NSW Australia; ^5^ School of Public Health University of Sydney Sydney NSW Australia; ^6^ Health Economics Group Norwich Medical School University of East Anglia Norwich UK; ^7^ Menzies Centre for Health Policy University of Sydney Sydney NSW Australia; ^8^ Discipline of Public Health School of Health Sciences Faculty of Medicine, Nursing and Health Sciences Flinders University Adelaide SA Australia

**Keywords:** checklist, citizen jury, CJCheck, community jury, reporting standards

## Abstract

**Background:**

Opportunities for community members to actively participate in policy development are increasing. Community/citizen's juries (CJs) are a deliberative democratic process aimed to illicit informed community perspectives on difficult topics. But how comprehensive these processes are reported in peer‐reviewed literature is unknown. Adequate reporting of methodology enables others to judge process quality, compare outcomes, facilitate critical reflection and potentially repeat a process. We aimed to identify important elements for reporting CJs, to develop an initial checklist and to review published health and health policy CJs to examine reporting standards.

**Design:**

Using the literature and expertise from CJ researchers and policy advisors, a list of important CJ reporting items was suggested and further refined. We then reviewed published CJs within the health literature and used the checklist to assess the comprehensiveness of reporting.

**Results:**

CJCheck was developed and examined reporting of CJ planning, juror information, procedures and scheduling. We screened 1711 studies and extracted data from 38. No studies fully reported the checklist items. The item most consistently reported was juror numbers (92%, 35/38), while least reported was the availability of expert presentations (5%, 2/38). Recruitment strategies were described in 66% of studies (25/38); however, the frequency and timing of deliberations was inadequately described (29%, 11/38).

**Conclusions:**

Currently CJ publications in health and health policy literature are inadequately reported, hampering their use in policy making. We propose broadening the CJCheck by creating a reporting standards template in collaboration with international CJ researchers, policy advisors and consumer representatives to ensure standardized, systematic and transparent reporting.

## 1 Background

It is incumbent on researchers to provide adequate descriptions of their research methodology and methods when reporting their findings in the peer‐reviewed literature. Where research methods are well described, we better understand the process by which the findings have been generated and have more confidence in appraising the credibility of these outcomes. Reporting standards also create an incentive for researchers to be meticulous in designing studies. Reporting guidelines have been developed for a range of methods and disciplines for these reasons (e.g. CONSORT, PRISMA, STARD,^1^ CHEERS^2^) and some journals now require these checklists to be completed prior to publication. More recently, templates have been developed to provide guidance for describing the interventions tested in research (TIDieR^3^). When used, these checklists allow comparisons between studies, enhance transparency, support trust in the process and provide robust foundations for future research.

In Western liberal democracies, there has been an increased openness in government public policy processes to include public voices in policy debate and formulation.^4^, ^5^, ^6^ Public engagement processes are gaining popularity, but their methods vary.^4^, ^7^ Three broad methods of public engagement include communicative (e.g. public meetings, website information), consultative (e.g. opinion poll, focus groups) and participatory (deliberative poll, citizen jury).^8^ This article is focused on a group of participatory, deliberative democratic processes with methodologies similar to those described by the Jefferson Center.^9^ Some have been called citizens’ panels, citizens’ fora and community/citizens’ jury. We refer to these, collectively, as community juries (CJs).

As a participatory form of public engagement, CJs aim to illicit an *informed* community perspective on controversial or difficult topics where the values and preferences of community members are sought. For this reason, CJ participants are recruited from the general population and deliberate on questions that require an ethically sensitive or value‐based decision. Common CJ elements include extensive provision of information from expert presentations, questioning opportunities, substantial time for deliberation and formulation of a consensus or majority “verdict” by CJ participants.^9^

CJs have been used to explore community perspectives on several health areas including screening,^10^, ^11^, ^12^ resource priority setting,^13^, ^14^ informed consent processes for screening decisions^15^ and pandemic planning.^16^ These particular examples were conducted for research purposes and none formally linked CJ outcomes to policy decisions. But in Australia, the extent to which CJs are embedded in policy processes is changing. Recently, CJs have been used by local and provincial governments to garner public input to policy making.^17^, ^18^, ^19^ However, despite the increased use in policy decision making, some hesitancy exists around the trustworthiness of CJ processes.^20^ If the process methods of CJs were reported comprehensively, consistently and transparently, their use in shaping health policy may increase.

Over a decade has passed since Abelson et al.^4^ suggested design and evaluation principles for public deliberations. Four key components were identified: representation of citizenry, documentation of deliberative procedures, descriptions of information provided to community members and reporting of outcomes. However, inconsistencies in reporting of these components still occur. A recent systematic review explored the adaptations in CJ methodologies and reported wide variations in how CJs were conducted and reported.^21^ Also, in a review of public deliberation in health policy, Abelson et al.^5^ continued to find inconsistencies and ambiguity in reporting method descriptions. Different CJs could legitimately have different outcomes even if they followed the same methodology (random recruitment, same expert information, etc.), but poor and/or inconsistent reporting means it is difficult to compare CJ findings or consider potential reasons for differing results, and it is not yet empirically clear how methodological variation may alter outcomes.

We cannot assess quality until we are clear about methodology. Implementers of CJs know little about what might enhance or detract from the legitimacy of claims about the representation of different types of public; quality of the processes of information provision and public deliberation; or the authenticity of the outcomes. We sought to determine the adequacy of process reporting in CJs published in the peer‐reviewed health and health policy literature. We used two processes. First, we conducted a Delphi survey with a group of published CJ researchers to generate an agreed checklist of CJ characteristics and research methods that are important to report in publications. Second, using this checklist, we then reviewed published CJs to establish the utility of the checklist and determine the most frequently reported and missing descriptions of the CJ methods.

## 2 Methodology

### 2.1 Stage 1: initial CJCheck development

Fourteen published Australian CJ researchers and selected government policy advisors attended a CJ Research Symposium at Bond University, Australia, in July 2015. Participants were invited if they were active Australian CJ researchers (13 invited, 10 attended); active policy advisors/government workers (four invited, all attended or were represented); and/or consumer representatives (two invited and neither able to attend). Symposium goals were broad with an aim to discuss several key research and policy questions. Outcomes included the identification of several research agenda and collaborative links. One research question arising from the symposium was to identify potentially important CJ characteristics and processes and determine whether these were reported in published CJ studies. Methodological elements of CJs were brainstormed and iteratively developed by all symposium participants in small group, round robin exercises. A smaller self‐selected working party (from the larger symposium group) participated in a two‐stage Delphi method^22^ to refine these CJ elements.

The first Delphi round was conducted in September 2015. The survey consisted of the 17 reportable methods suggested by the larger CJ symposium group. These criteria were divided into four groups (planning the CJ, characteristics of the jurors, CJ procedures and CJ schedule). Respondents were asked to rate each criterion on a five‐point Likert scale (1=“important” to 5=“unimportant”). Changes to item wording and new items could be suggested for ranking in the second round. Items that were scored by six or more respondents as “important” or “somewhat important” were retained. Items that had fewer than five respondents scoring in these top two categories were re‐ranked in the second round.

The second round was conducted in October 2015. Questions were again ranked on a five‐point Likert scale. As before, there was one optional, free response question, for further suggestions. On this occasion, respondents were also asked whether the item was important but not necessary to report.

The final checklist (CJCheck) was used in data extraction. Seven items were rated as either “yes” or “no,” while the remainder as “yes,” “no” or “unclear.” Specific checklist criteria are reported in Table S1.

### 2.2 Stage 2: selection of CJ studies and data extraction

We used two strategies to search for included studies. First, included articles were drawn from two recent systematic reviews pertaining to deliberative democracy.^21^,^23^ These systematic reviews included a broad range of deliberative democratic techniques; therefore, we only included articles from these reviews that nominated the deliberative democratic technique most resembling CJs (e.g. random or representative selection of jurors, provision of balanced information to jurors, sufficient time allocated to deliberation).^8^ Included studies from these two reviews described the deliberative process as “citizens’ panels,” “community or citizens’ juries,” “citizens’ council” or “citizens’ fora”).

In addition to including relevant studies from the two previous reviews, we replicated the search strategy of Degeling et al.^23^ and modified the search dates to encompass 2013 to 2015 to include studies published since the initial search. We conducted the new search in *Medline*,* Web of Science*,* Current Contents Connect* and *Scopus* databases in September 2015. Therefore by combining the two search strategies, we included published CJ studies in the health and health policy literature between 1996 and September 2015.

Remaining consistent with others,^9^, ^21^, ^23^ we included studies that described the deliberative process as having characteristics similar to CJs. Our inclusion criteria required studies to describe any or some characteristics resembling those outlined by the Jefferson Centre^9^ regardless of whether they were reported in detail. For example, studies were required to define themselves as conducting a citizen jury, community jury, citizen panel or fora, providing information to participants from different “experts” and having a deliberative component.^9^ Protocols were excluded. All studies were screened independently against eligibility criteria by two authors (R.T. and R.S.). Conflicts were checked periodically and resolved through discussion. Criteria definitions were clarified where necessary.

### 2.3 Data analysis

Data extraction was conducted by the same two authors using the checklist developed in the Delphi rounds. Data were analysed descriptively using Excel.

## 3 Results

### 3.1 Stage 1: initial CJCheck development

In the first Delphi round, 13 CJ methodology reporting criteria were identified as either important or somewhat important and retained. In the second Delphi round, nine items were presented: four from round one that required further ranking and five new items. Following the second round, a further three questions were indicated to be important in the reporting of CJs and were retained, two were combined with other retained items, one item was reworded and retained and three were excluded. The Delphi round outcomes are reported in Table 1. No items were consistently ranked as “somewhat unimportant” or “unimportant.”

**Table 1:** Ranking outcomes of Delphi rounds 1 and 2

	Checklist items	Ranking of Delphi respondents					
		Important	Somewhat important	OK	Somewhat unimportant	Unimportant	Important, but not necessary to report
Round 1 (N=8)							
Planning	Was the stakeholder/committee's role clearly described?	4	2	1	1	0	NA
	Was the selection of experts (who was chosen and why) adequately described?	4	3	1	0	0	NA
	Were the experts roles clearly defined?	5	3	0	0	0	NA
	Was the Jury “charge” or instruction clearly described?	6	2	0	0	0	NA
Jurors	Was the study recruitment strategy clearly described?	6	2	0	0	0	NA
	Were inclusion/exclusion criteria reported?	7	1	0	0	0	NA
	Was the type of participant/juror described (unaffected public, affected public, advocate)?	6	1	1	0	0	NA
	Were the demographics of the jurors reported (age, gender, education, attainment)?	3	4	1	0	0	NA
Procedure	Was the role and experience of the facilitator described (e.g. impartial, informed, member of research team, independent)?	2	2	4	0	0	NA
	Were materials provided to the jurors adequately described and accessible?	5	2	1	0	0	NA
	Was the expert cross‐examination opportunities described?	2	5	1	0	0	NA
	Was the jury outcome reported?	6	0	1	0	1	NA
Scheduling	Was the schedule (how often and interval) and length (days/hours) of juror meetings reported?	6	2	0	0	0	NA
	Was the daily schedule of events described?	1	4	3	0	0	NA
	Was the number of presenters and their topics described?	4	2	2	0	0	NA
	Are the expert presentations available?	2	3	3	0	0	NA
	Were the lengths of the presentations reported?	0	4	2	2	0	NA
New items suggested from Round 1	Were the jurors paid?	–	–	–	–	–	NA
	How many jurors were there?	–	–	–	–	–	NA
	What was the influence of the jury outcome on policy?	–	–	–	–	–	NA
	What was the framing/nature of jury deliberations?	–	–	–	–	–	NA
	What was the influence of the commissioning body on the jurors?	–	–	–	–	–	NA
Round 2 (N=9)							
Re‐ranked from Round 1	Was the role and experience of the facilitator described (e.g. impartial, informed, member of research team, independent)?	3	4	0	2	0	0
	Was the schedule (how often and interval) and length (days/hours) of juror meetings reported?	4	4	1	0	0	0
	Are the expert presentations available?	3	4	1	0	0	1
	Were the lengths of the presentations reported?	2	1	5	0	0	1
New items suggested from Round 1: to rank	Were the jurors paid?	2	1	3	3	0	0
	How many jurors were there?	6	2	1	0	0	0
	What was the influence of the jury outcome on policy?	1	3	1	2	1	1
	What was the framing/nature of jury deliberations?	5	1	2	0	0	0
	What was the influence of the commissioning body on the jurors?	2	3	0	2	1	1

Six checklist items were rated higher in importance than others. To be designated as an important item, it had to be rated either important or somewhat important by a clear majority of respondents (8/8 respondents in the first round and 8/9 respondents in the second round).

The final checklist contained 17 items grouped into four areas: planning of the CJ; juror information and characteristics; procedural information; and the scheduling of CJs.

### 3.2 Stage 2: adequacy of CJ method reporting

**Figure 1:**
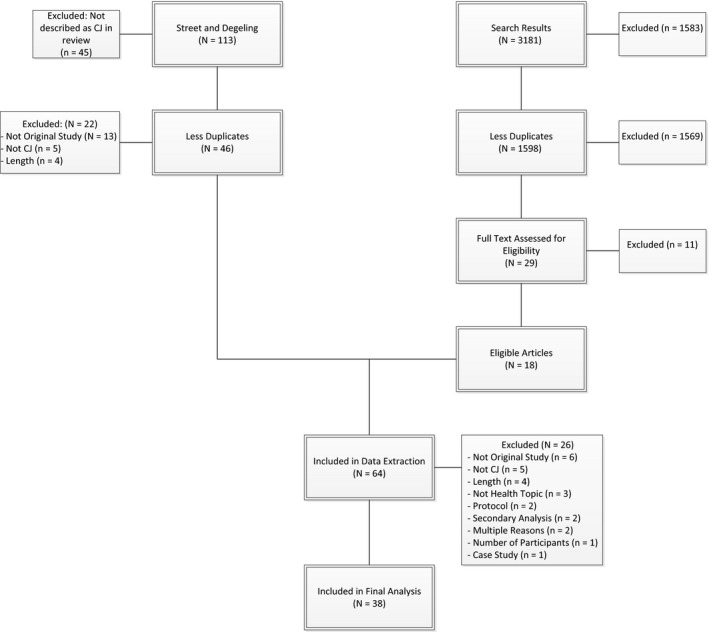
PRISMA flow chart

From the two systematic reviews,^21^, ^23^ 45 studies were excluded because they were not described as a CJ and 22 studies were reported in both reviews, leaving a total of 46 studies for full‐text review. In addition to these, our focused literature search modified from Degeling et al.^23^ found 1598 de‐duplicated articles for further analysis, of which only 18 met the inclusion criteria and were included in full‐text review and data extraction. We excluded 26 full‐text articles from data extraction, leaving 38 studies in our final analyses (see Fig. 1). Included studies and their characteristics are listed in Table 2, with data extraction details of included studies available in Table S2 and reason for study exclusion from full‐text review provided in Table S3.

**Table 2:** Studies included in data extraction

Article information	Health topic	Charge
Bennett et al., 2007,^26^ Genetics, insurance and participation: how a citizens’ jury reached its verdict	Genetic Test Results and Life Insurance	Consider three policy options that might follow the expiry of the moratorium on the use of genetic tests by insurers.
Bombard et al., 2011,^27^ Eliciting ethical and social values in health technology assessment: A participatory approach	Health technologies	What are the core values that should guide MASOHTAC evaluations of health technologies, when and by whom should this be done?
Bombard et al., 2013,^28^ Citizens’ perspectives on personalised medicine: a qualitative public deliberation study	Health technologies assessment	What questions do you have about the value of GEP that you would like to see AHTAC include in its review?What questions do you have about the area of personalized medicine, in general, that OHTAC could consider in its review of these new technologies? Which ethical and social questions from Hofmann's list need to be applied to (i) GEP and (ii) personalized medicine in general?
Braunack‐Mayer et al., 2010,^16^ Including the public in pandemic planning: a deliberative approach	Pandemic planning	Who should be given the scarce antiviral drugs and vaccine in an influenza pandemic? Under what circumstances would quarantine and social distancing measures be acceptable in an influenza pandemic?
Burgess et al., 2008,^24^ Biobanking in British Columbia: discussion of the future of personalised medicine through deliberative public engagement	Biobanking	Main goals of each day were to share knowledge, identify values and design a biobank
Carman et al., 2015,^29^ Effectiveness of public deliberation methods for gathering input on issues in healthcare: results from a randomised trial	Medical evidence determining healthcare choices	Should individual patients and/or their doctors be able to make any health decisions no matter what the evidence of medical effectiveness shows, or should society ever specify some boundaries for these decisions?
Chafe et al., 2010,^30^ Does the public think it is reasonable to wait for more evidence before funding innovative health technologies? The case of PET scanning in Ontario	Health technologies	In your opinion, is the Ontario Ministry of Health and Long‐Term Care's (MOHLTC) approach to funding PET scanning a reasonable one and in the nest interests of the citizens of Ontario? What suggestions for modifications do you have, and why?
Chafe et al., 2011,^31^ Accepting new patients. What does the public think about Ontario's policy?	Accepting new patients	Do you think that the CPSO policy “Accepting New Patients” is reasonable? If yes, how would you make it better? If not, what fundamental changes should be made?
Dunkerley et al., 1998,^32^ Empowering the public? Citizens’ juries and the new genetic technologies	Introduction of new genetic technologies into health care	What conditions should be fulfilled before genetic testing for people susceptible to common diseases becomes available on the NHS?
Einsiedel & Ross, 2002,^33^ Animal spare parts? A Canadian public consultation on Xenotransplantation	Xenotransplantation	The safety, efficacy, ethical and regulatory issues surrounding the potential use of xenografts
Einsiedel, 2002,^34^ Assessing a controversial medical technology: Canadian public consultations on xenotransplantation	Xenotransplantation	Should Canada proceed with xenotransplantation and if so, under what conditions?
Elwood & Longley, 2010,^35^ My health: whose responsibility? A jury decides	Where does the responsibility for maintaining a person's health lie	What is “illness” and what is “health” and what is the overlap between these? What are my responsibilities, and those of others, in maintaining my health? What help should I expect when making decisions about my health? Who knows best – the public, general practitioners or hospital specialists, and to what extent, and in what manner, should I, as a member of the general public, be informed? How should the risks and benefits of medicines be balanced in relation to the prevention of disease and the maintenance of health? Who should evaluate this balance and who should take the decisions about medicines and behaviours which will help maintain my health? What is the role of the regulatory authorities?
Finney, 2000,^36^ Implementing a citizen‐based deliberative process on the internet: the Buckinghamshire health authority electronic citizens’ jury in the UK	Management of back pain	Should Buckinghamshire Health Authority fund treatment from osteopaths and chiropractors for people with back pain?
Fish et al., 2014,^37^ Employing the citizens’ jury technique to elicit reasoned public judgments about environmental risk: insights from an inquiry into the governance of microbial water pollution	Microbial water pollution	What risks arise from the microbial pollution of water courses and how significant are they? What are the origins of these microbial risks and how culpable are livestock farming practices within them? What more could reasonable be done to mitigate the impact of livestock farming practices on water quality? Where do responsibilities begin and end when controlling these microbial risks arising from livestock farming?
Gooberman‐Hill et al., 2008,^13^ Citizens’ juries in planning research priorities: process, engagement and outcome	Local priorities for health and social care research	What are the priorities of the citizens of Bristol for research into the provision of primary health and social care?
Herbison et al., 2009,^38^ Research priorities in urinary incontinence: results from citizens’ juries	Urinary incontinence	What can researchers study to make your life better?
Hodgetts et al., 2014,^39^ Disinvestment policy and the public funding of assisted reproductive technologies: Outcomes of deliberative engagements with three key stakeholder groups	Assisted reproductive technologies	Should the criteria for public funding of ART be changed? If yes, why? If no, why not?
Iredale et al., 1999,^40^ Public involvement in policy making: the case of a citizens’ jury on genetic testing for common disorders	Genetic Testing	What conditions should be fulfilled before genetic testing for susceptibility to common disorders becomes widely available on the NHS?
Iredale et al., 2006,^41^ What choices should we be able to make about designer babies? A Citizens’ jury of young people in South Wales	Technology for reproducing decision making	Designer babies: What choices should we be able to make?
Kashefi et al., 2004,^42^ Grounded citizens’ juries: a tool for health activism?	Improvement of health and well‐being	What would improve the health and well‐being of residents of SWB?
Lee et al., 2014,^43^ Technology and citizens an analysis of citizens’ jury on the Korean national pandemic response system	Pandemic Response	What is the likelihood of a national pandemic occurring in Korea due to the avian influenza? How would you rate Korea's response system against a possible outbreak of a national pandemic? What are the areas of improvement necessary to ensure effective readiness and response against a national pandemic? What are the ways of enhancing citizens’ understanding and confidence in the National Response System?
Lenaghan et al., 1996,^44^ Setting priorities: Is there a role for citizens’ juries?	Priority setting	How priorities for purchasing health care should be set, according to what criteria, and what role, if and, the public should have in these decision
Longstaff & Burgess, 2010,^45^ Recruiting for representation in public deliberation on the ethics of biobanks	Biobanking	–
McWhirter et al., 2014,^46^ Community engagement for big epidemiology: deliberative democracy as a tool	Biobanking	–
Menon et al., 2008,^47^ Engaging the public in priority‐setting for health technology assessment: findings from a citizens’ jury	Setting priorities for health technology assessment	Developing a set of criteria to guide priority setting of HTA
Molster et al., 2013,^48^ Blueprint for a deliberative public forum on biobanking policy: Were theoretical principles achievable in practice?	Biobanking	Develop recommendations on how biobanking should be set up and operate in Western Australia
Moretto et al., 2014,^49^ Yes, the government should tax soft drinks: Findings from a citizens’ Jury in Australia	Taxation of junk food	Is taxation on food and drinks an acceptable strategy to the public in order to reduce rates of childhood obesity?
Nep et al., 2013,^50^ Understanding Public Calls for Labeling of Genetically Modified Foods: Analysis of a Public Deliberation on Genetically Modified Salmon	Genetically modified foods	Should the salmon genome be sequenced? Why or why not?
O'Doherty & Burgess, 2009,^51^ Engaging the public on biobanks: outcomes of the BC biobank deliberation	Biobanking	Design specifications for biobanking in British Columbia
Parkin & Paul, 2009,^52^ Public good, personal privacy: a citizens’ deliberation about using medical information for pharmacoepidemiological research	Use of medical information	Should researchers contracted by a public body be permitted to use medical information about identifiable people, without their consent, for the following purposes: routine analysis to identify the potential adverse effects of medicines that are newly introduced into New Zealand; investigation of emerging concerns about the adverse effects of medicines currently being used by New Zealanders? If so, under what circumstances and with what safeguards, if any? If not, why?
Paul et al., 2008,^11^ Making policy decisions about population screening for breast cancer: the role of citizens’ deliberation	Breast Cancer Screening	Should the New Zealand government offer free screening mammograms to all women aged 40–49 years?
Rogers et al., 2009,^53^ Pandemic influenza communication: views from a deliberative forum	Pandemic Influenza Planning	What is an acceptable framework for communication in an influenza pandemic?
Rychetnik et al., 2014,^54^ A Community Jury on PSA screening: what do well‐informed men want the government to do about prostate cancer screening—a qualitative analysis	PSA screening	Deliberate evidence presented by experts on PSA screening and formulate recommendations on potential government actions
Secko et al., 2009,^55^ Informed consent in biobank research: a deliberative approach to the debate	Biobanking	–
Stafinski et al., 2014,^56^ Assessing the impact of deliberative processes on the views of participants: Is it in one ear and out the other?	Health technologies	–
Thomas et al., 2014,^10^ Deliberative democracy and cancer screening consent: a randomized control trial of the effect of a community jury on men's knowledge about and intentions to participate in PSA screening	PSA screening	What do you, as a group of men, think about a government organized invitation programme for testing for prostate cancer?
Timotijevic et al., 2007,^57^ Evaluation of two methods of deliberative participation of older people in food‐policy development	Food Policy	Does food retailing need to change in order to achieve optimal health and diet?
Toni et al., 2001,^58^ Poor citizens decide on the introduction of GMOs in Brazil	Genetically modified organisms	Can GMOs contribute to solving hunger in Brazil and the world? Can GMOs facilitate access to food and food security and serve the interests of small‐scale farmers and the poor? Does enough scientific evidence exist about the consequences of GMOs for the environment to justify their release? Has the analysis, monitoring, reporting and decision making on field trials and commercial liberalization been done with sufficient caution, transparency and participation of civil society? Is there sufficient information on GMOs and is it accessible? Can consumers and farmers exercise a right to choose?

**Figure 2:**
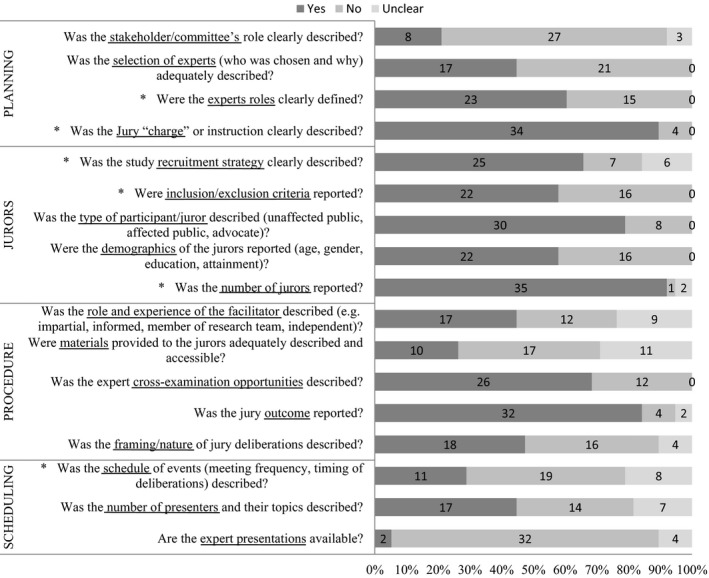
The number of included studies with rating descriptions of checklist items. *Indicates items identified by Delphi respondents as important

Studies varied widely in their reporting of the methodological processes necessary for reliable CJ reproduction. No study reported all 17 checklist items. On average, only 53% of checklist items (nine items from a possible 17) were reported in any given article (SD=19.5%) and this proportion ranged from 12% (2/17 items^13^) to 88% (15/17 items^24^). Overall, the checklist item reported most consistently was “Was the number of jurors reported” (92%, 35/38 studies). In contrast, the least reported item was “Are the expert presentations available,” which was only reported in 5% (2/38 studies) of articles. Figure 2 shows the proportion of articles reporting checklist items.

### 3.3 Planning the CJ

When reporting about planning a CJ, 21% (8/38) of studies described whether they had a stakeholder group or committee and if so, the role of this group in CJ planning. Less than half (45%, 17/38) stated how and why the experts were chosen; however, more (58%, 22/38) defined the roles of the experts and most (89%, 34/38) gave a clear description of the jury “charge.”

### 3.4 Information about the jurors

The majority of studies reported some type of juror information. The recruitment strategy was described in 66% (25/38) of studies and inclusion and exclusion criteria were described in 58% (22/38). Whether the juror was an affected member of the public, unaffected member of the public, advocate or invited via random sampling was reported in 79% (30/38) of studies. However, only just over half (58%, 22/38) reported the demographic characteristics of the jurors. The most consistently reported juror detail was the number of jurors (92%, 35/38).

### 3.5 Procedural information

There was a wide variation in what procedural information was provided in the published studies. The role and experience of the facilitator was described in less than half (45%, 17/38) of the examined studies. There was an absence of description and accessibility of materials provided to the jurors, with this reported in only 26% (10/38) of studies. Approximately 68% (26/38) described the expert cross‐examination opportunity provided to the jurors. The outcome of juries was the most consistently reported criterion in this section with 84% (32/38) reporting this outcome. But only 47% (18/38) described how the jurors were instructed to deliberate.

### 3.6 CJ scheduling information

Overall, CJ scheduling information was poorly reported. The frequency and timing of deliberations was reported in only 29% (11/38) of examined studies. The number of presenters and their topics was reported less than half the time (45%, 17/38) and the availability of expert presentations was reported in only 5% of studies (2/38).

## 4 Discussion

Overall, our findings indicate considerable opportunity for improvement in the reporting of CJs. No published CJs we assessed fully described CJCheck items identified by CJ researchers as important to report. Less than half of the studies in our analyses reported the role of the stakeholder committee; how and why experts were selected; the role of the facilitator; a description of the materials provided to the jurors; how the deliberations were framed; the scheduling of events; the number of presenters and their topics; and whether the expert presentations were available. The most reported checklist items were the number of jurors, a description of the jury charge and the jury outcome. Although these are essential reporting items, they are not sufficient.

Although in this review we have focused on peer‐reviewed publications, we believe our suggested checklist items apply to all reported CJs. CJ organizers need to be sufficiently transparent and rigorous in reporting their methods for several reasons: leaving open the possibility of attempting to approximate and repeat a CJ elsewhere; enabling judgements about the quality of the CJ process; allowing comparison of juries; and facilitating critical reflection on CJ design and its potential effect on CJ outcomes. We cannot do this if authors inconsistently report methodology and methods.

It is not our contention that if a particular CJ was repeated that the CJ “verdict” and reasoning would necessarily be the same. It is feasible that it might not be, given different contexts and participants and therefore different values and preferences. But it is also feasible that repeating a CJ, with different participants but the same content and processes, may produce similar outcomes. Because CJ methods are not currently consistently reported, we cannot assess these questions. Perhaps more importantly, comprehensive reporting of CJs supports transparency and would permit the improved evaluation of CJ methodologies. How representative, procedurally fair and accountable CJs are in the health sector has not been fully evaluated.^4^, ^21^ This hampers the utility of CJs to contribute to important health policy debates.

Our study has numerous strengths and some limitations. We conducted a focused review of published CJ studies within the health sector and mapped descriptions of their methods to criteria developed by CJ researchers. The criteria checklist had two rounds of iterations by published CJ authors to ensure that important methodology and processes were captured. Studies were then independently rated using these criteria for completeness of reporting. However, the criteria may be somewhat limited as they were developed by a group of Australian CJ researchers and policy advisors and may not reflect the wider view of other CJ organizers. We consider this an important future development. Additionally, we rated the adequacy of reporting on the basis of the written publication and did not contact the authors for further information. This was a deliberate choice, as our purpose was to assess the adequacy of reporting of published CJs.

Other CJ research has reported the variations in methodology.^5^, ^21^, ^23^ This was the first study to quantify those variations by mapping researcher‐identified methodological reporting criteria. Our process was similar to the initial development of the TIDieR checklist,^25^ the uptake of which seems likely to strengthen the utility of intervention research. We intend to expand our consortium of CJ researchers, policy advisors and consumer representatives to develop an internationally agreed CJ reporting template. We expect that the legitimization of CJs’ input into public health policies will be assisted by the use of standards for reporting of methodology and methods.

## 5 Conclusion

To fully report processes within a CJ, it is important that the planning of the event, information about the jurors and their recruitment, and procedural and technical information be available and clearly documented. Our findings suggest that many current CJ publications in health and health policy literature are inadequately reported. Therefore, methods are not transparent and readers cannot compare CJ processes and outcomes. This may not be solely a result of inadequate reporting, but also due to the absence of specific reporting standards for CJs.

Street et al.^21^ suggested that strict adherence to CJ methodology as described by the originators might limit the generation of new knowledge. We agree. We advocate that CJ organizers and authors be committed to rigour and openness, but also innovation. It is through testing and adapting methodologies that new ideas are developed and understanding expands. As researchers who utilize CJ methodology, it is not our intention to restrict methodological development, but to enhance understanding. In this study, we present an empirically developed and trialled, proposed checklist for reporting CJ methodology and methods. We advocate broadening CJCheck by creating a reporting standards template developed by a consortium of international CJ researchers, health policy advisors and consumer representatives to use when designing and reporting CJs.

## Conflict of Interest

The authors report no conflicts of interest.

## Funding Support

RT was supported by a NHMRC Screening and Test Evaluation Program (STEP) Grant (#633033). RS was supported by a Bond University Vice Chancellor's Research Grant Scheme. CD, SMC and LR received funding support from NHMRC Project Grant (#1023197). CD received funding support from a NHMRC Project Grant (#1083079). SMC is funded through NHMRC Career Development Fellowship (#1032963). JMS was funded by an Australian National Preventive Health Agency Fellowship (20STR2013F) and an NHMRC Capacity Building Grant (565501).
