# Supplementary material for: CJCheck Stage 1: development and testing of a checklist for reporting community juries – Delphi process and analysis of studies published in 1996–2015
Source: Health Expect. 2016 Oct 5;20(4):626–37. doi: 10.1111/hex.12493 (PMC5513001; doi:10.1111/hex.12493)
Supplement: Supplementary file 1 [file HEX-20-626-s001.docx]

**Supplementary Table 1. Item Checklist for use in Data Extraction**

| **Checklist Item** | **Question** | **Explanation** | **Checklist Responses** | | |
| --- | --- | --- | --- | --- | --- |
|  |  |  | **Yes** | **No** | **Unclear** |
| Planning | Was the stakeholder/committee’s role clearly described? | Who are the stakeholders and what is their role/influence on the CJ? | Reported and clear | Not reported | Vague description, not clear; may be available from authors |
|  | Was the selection of experts (who was chosen and why) adequately described? | Who are the experts and why were they chosen? | Reported and clear | Not reported | NA |
|  | Were the experts roles clearly defined? | What were the experts required to do (presentations, Q&A)? | Reported and clear | Not reported | NA |
|  | Was the Jury “charge” or instruction clearly described? | Why was the jury conducted (to influence policy, decision making, inform) and did they have a clear question to answer? | Reported and clear | Not reported | NA |
| Jurors | Was the study recruitment strategy clearly described? | Is the description sufficient to reproduce the recruitment strategy? | Reported and clear | Not reported | Vague description, not clear; may be available from authors |
|  | Were inclusion/exclusion criteria reported? | Were the inclusion/exclusion criteria outlined? | Reported and clear | Not reported | NA |
|  | Was the type of participant/juror described (unaffected public, affected public, advocate)? | Were the juror’s relationship to the problem described e.g, unaffected public(naïve citizen), affected public (individuals with condition), advocate of the topic in question (representatives of affected public)? | Reported and clear; also if random sampling was used | Not reported | NA |
|  | Were the demographics of the jurors reported (age, gender, education, attainment)? | Were the relevant demographic characteristics of the jurors described? (e.g., age, gender, education, etc)? | Reported and clear | Not reported | NA |
|  | Was the number of jurors reported? | How many jurors attended the CJ event? | Reported | Not reported | If multiple CJs, total number provided but not breakdown; may be available from authors |
| Procedure | Was the role and experience of the facilitator described (e.g. impartial, informed, member of research team, independent)? | Was the facilitator described as impartial, informed, a member of the research team, independent? | Reported and clear | Not reported | Either role or experience described but not both |
|  | Were materials provided to the jurors adequately described and accessible? | Can you download these/is the description comprehensive? | Materials are clearly described and/or accessible (referenced) | Not reported | Materials reported but not clearly described; may be available from authors |
|  | Was the expert cross-examination opportunities described? | Were the jurors able to cross-examine the witnesses/experts? | Reported and clear | Not reported | NA |
|  | Was the jury outcome reported? | Is the final outcome of the jury detailed? | Reported and clear | Not reported | Some outcomes reported but incomplete; may be available from authors |
|  | Was the framing/nature of jury deliberations described? | Was the method of jury deliberation described (guided, free, set questions) | Reported and clear | Not reported | Vague description, not clear |
| Scheduling | Was the schedule of events (meeting frequency, timing of deliberations) described? | Was the scheduling of events sufficiently reported to allow others to repeat the timings (e.g., event breakdown, length of presentations, and deliberations? | Detailed description of daily sessions /activities | Not reported | Broad description of sessions/activities, not detailed; may be available from authors |
|  | Was the number of presenters and their topics described? | How many experts presented and on what topics? | Presenters or professions and topics described | Not reported | Either presenters/professions or topics described but not both; may be available from authors |
|  | Are the expert presentations available? | Are the presentations stated as being available for download or from the author? | Reported and available | Not reported | Appears to be in a format that is available but not clear; may be available from authors |
