# Supplementary material for: CJCheck Stage 1: development and testing of a checklist for reporting community juries – Delphi process and analysis of studies published in 1996–2015
Source: Health Expect. 2016 Oct 5;20(4):626–37. doi: 10.1111/hex.12493 (PMC5513001; doi:10.1111/hex.12493)
Supplement: Supplementary file 2 [file HEX-20-626-s002.docx]

**Supplementary Table 2. Checklist Criteria and Data Extraction**

|  | **Planning** | | | | **Jurors** | | | | | **Procedure** | | | | | **Schedule** | | |
| --- | --- | --- | --- | --- | --- | --- | --- | --- | --- | --- | --- | --- | --- | --- | --- | --- | --- |
| Article Information | **Stakeholder** | **Expert Selection** | **Expert Role** | **Charge** | **Recruitment** | **Inclusion Criteria** | **Type of Participant** | **Demographics** | **Number of Jurors** | **Facilitator** | **Materials** | **Cross-examination** | **Outcome** | **Framing** | **Schedule of Events** | **Presenters** | **Expert Presentations** |
| Bennett et al., 2007 | N | N | Y | Y | Y | Y | Y | Y | Y | U | N | Y | Y | Y | U | U | N |
| Bombard et al., 2011 | N | N | N | Y | Y | N | Y | Y | Y | U | U | Y | Y | Y | N | N | N |
| Bombard et al., 2013 | N | N | N | Y | N | N | Y | Y | Y | U | U | Y | Y | Y | N | N | N |
| Braunack-Mayer et al., 2010 | N | N | N | Y | N | N | N | N | Y | Y | U | N | Y | Y | N | N | N |
| Burgess et al., 2008 | Y | N | Y | Y | Y | Y | Y | Y | Y | Y | Y | Y | Y | Y | Y | Y | U |
| Carman et al, 2015 | N | N | Y | Y | U | Y | N | N | Y | N | Y | Y | Y | U | N | N | N |
| Chafe et al., 2010 | N | N | Y | Y | N | Y | Y | N | Y | Y | N | Y | Y | Y | Y | Y | N |
| Chafe et al., 2011 | N | N | Y | Y | N | Y | Y | N | Y | Y | N | Y | Y | Y | Y | Y | N |
| Dunkerley et al., 1998 | Y | N | N | Y | U | N | Y | N | Y | Y | N | Y | U | N | Y | U | N |
| Einsiedel & Ross, 2002 | Y | Y | Y | Y | N | N | N | N | N | U | U | Y | Y | Y | Y | Y | N |
| Einsiedel, 2002 | Y | Y | Y | Y | U | N | N | N | U | U | N | Y | Y | Y | U | Y | N |
| Elwood &  Longley, 2010 | U | N | Y | Y | Y | Y | Y | N | Y | U | N | Y | Y | N | N | N | N |
| Finney, 2000 | N | Y | N | Y | N | N | N | N | U | N | Y | N | N | N | Y | Y | U |
| Fish et al., 2014 | N | Y | Y | Y | Y | Y | Y | Y | Y | Y | U | Y | Y | U | N | Y | N |
| Gooberman-Hill et al., 2008 | N | N | N | Y | U | N | N | N | Y | U | N | N | U | N | N | N | N |
| Herbison et al., 2009 | N | Y | Y | Y | Y | Y | Y | Y | Y | N | N | Y | Y | U | Y | Y | N |
| Hodgetts et al., 2014 | N | N | N | Y | U | N | Y | N | Y | Y | N | N | Y | N | N | N | N |
| Iredale et al., 1999 | N | Y | Y | Y | Y | Y | Y | Y | Y | Y | U | Y | Y | N | N | U | N |
| Iredale et al., 2006 | Y | N | Y | Y | Y | Y | Y | Y | Y | Y | U | Y | Y | N | U | U | Y |
| Kashefi et al., 2004 | Y | Y | N | Y | U | N | Y | Y | Y | N | U | N | Y | N | N | N | N |
| Lee et al., 2014 | Y | N | N | Y | Y | Y | Y | Y | Y | N | N | Y | Y | U | N | N | N |
| Lenaghan et al., 1996 | N | Y | Y | Y | Y | N | Y | N | Y | Y | N | N | Y | N | N | Y | N |
| Longstaff & Burgess, 2010 | N | N | N | N | Y | Y | Y | Y | Y | U | N | N | N | N | U | U | N |
| McWhirter et al., 2014 | N | Y | Y | N | Y | Y | Y | Y | Y | Y | Y | N | Y | Y | Y | Y | N |
| Menon et al., 2008 | N | N | Y | Y | Y | Y | Y | Y | Y | Y | N | Y | Y | Y | Y | N | N |
| Molster et al., 2013 | N | Y | Y | Y | Y | N | Y | Y | Y | Y | U | Y | N | Y | U | N | N |
| Moretto et al., 2014 | N | Y | Y | Y | Y | N | Y | Y | Y | Y | U | Y | Y | Y | Y | Y | N |
| Nep et al., 2013 | N | N | N | Y | Y | N | Y | Y | Y | Y | Y | N | Y | Y | N | N | N |
| O'Doherty & Burgess, 2009 | N | Y | Y | Y | Y | Y | Y | N | Y | N | N | Y | Y | Y | U | Y | U |
| Parkin & Paul, 2009 | Y | Y | Y | Y | Y | N | Y | Y | Y | Y | Y | Y | Y | Y | U | Y | N |
| Paul et al., 2008 | N | Y | Y | Y | Y | Y | Y | Y | Y | N | Y | Y | Y | N | U | Y | N |
| Rogers et al., 2009 | U | N | N | Y | Y | Y | Y | Y | Y | Y | U | Y | Y | Y | N | N | N |
| Rychetnik et al., 2014 | N | Y | Y | Y | Y | Y | N | Y | Y | N | Y | Y | Y | Y | Y | Y | U |
| Secko et al., 2009 | N | N | N | N | Y | N | Y | N | Y | N | Y | N | Y | N | N | U | N |
| Stafinski et al., 2014 | N | N | N | N | Y | Y | Y | Y | Y | N | N | N | N | N | N | N | N |
| Thomas et al, 2014 | N | Y | Y | Y | Y | Y | N | Y | Y | N | Y | Y | Y | N | N | Y | Y |
| Timotijevic et al., 2007 | U | Y | Y | Y | Y | Y | Y | N | Y | N | N | N | Y | N | N | Y | N |
| Toni et al., 2001 | N | N | N | Y | N | Y | Y | N | Y | U | N | Y | Y | N | N | U | N |

Note. Y = Yes; N = No; U = Unclear
