# Supplementary material for: CJCheck Stage 1: development and testing of a checklist for reporting community juries – Delphi process and analysis of studies published in 1996–2015
Source: Health Expect. 2016 Oct 5;20(4):626–37. doi: 10.1111/hex.12493 (PMC5513001; doi:10.1111/hex.12493)
Supplement: Supplementary file 3 [file HEX-20-626-s003.docx]

**Supplementary Table 3. Studies Excluded from Full-Text Review**

| **Article Information** | **Reason for Exclusion** |
| --- | --- |
| Aasen et al., 2013, Deliberation on GMOs: A study of how a citizens' jury affects the citizens' attitudes | Not on health topic  (Genetically modified plants) |
| Abelson et al., 2003, Does deliberation make a difference? Results from a citizens panel study of health goals priority setting | Not CJ |
| Abelson et al., 2013, Assessing the impacts of citizen deliberations on the health technology process | Secondary Analysis |
| Brooks et at., 2003, Advancing end of life care: lessons learned from a statewide panel | Not Original Study;  Number of participants |
| Button & Mattson, 1999, Deliberative Democracy in Practice: challenges and prospects for civic deliberation | Not Original Study |
| Carson, 2006, Improving Public Deliberative Practice: A Comparative Analysis of Two Italian Citizen's Jury Projects in 2006 | Not Original Study |
| Comans et al., 2013, The cost effectiveness and consumer acceptability of taxation strategies to reduce rates of overweight and obesity among children in Australia: study protocol | Protocol |
| Docter et al., 2011, Public perceptions of pandemic influenza resource allocation: a deliberative forum using Grid/Group analysis | Secondary Analysis |
| Evers et al., 2009, Knowledge transfer from citizens' panels to regulatory bodies in the domain of nano-enabled medical applications | Not CJ |
| Farrell et al., 2013, Deliberative Democracy in Action Irish-style: the 2011 we the citizens pilot citizens assembly | Number of participants |
| Glasner et al., 1999, The new genetics, public involvement and citizens' juries: a welsh case study | Case Study |
| Haigh et al., 2008, Engaging communities to tackle anti-social behaviour: a health impact assessment of a citizens' jury | Not Original Study |
| Henderson et al., 2013, Evaluating the use of citizens' juries in food policy: a case study of food regulation | Length (3hrs) |
| Huston, 2004, What does the public think of placebo use? The Canadian experience | Length (1 day) |
| Kim et al., 2009, Assessing the public's views in research ethics controversies: deliberative democracy and bioethics as natural allies | Length (1 day) |
| Kim et al., 2010, Deliberative assessment of surrogate consent in dementia research | Length (1 day);  Number of participants |
| King et al., 2011, Exploring public perspectives on E health: findings from two citizen juries | Length (1 day) |
| Lenaghan, 1999, Involving the public in rationing decisions. The experience of citizens juries | Not Original Study |
| Lo et al., 2013, Reciprocity as deliberative capacity: lessons from a citizen's deliberation on carbon pricing mechanisms in Australia | Not CJ |
| Marsh et al., 2013, Consulting communities on feedback of genetic findings in international health research: sharing sickle cell disease and carrier information in coastal Kenya | Not CJ |
| Mooney et al., 2004, Whose health service is it anyway? Community values in healthcare | Not Original Study |
| Pidgeon et al., 2007, Opening up nanotechnology dialogue with the publics: risk communication of 'upstream engagement'? | Not on health topic (Nanotechnology) |
| Rogers-Hayden et al., 2006, Reflecting upon the UK's Citizens' jury on nanotechnologies: NanoJury UK | Not on health topic (Nanotechnology) |
| Pickard, 1998, Citizenship and consumerism in health care: a critique of citizens' juries | Not Original Study |
| Scuffham et al., 2014, Engaging the public in healthcare decision-making: quantifying preferences for healthcare through citizens' juries | Protocol |
| Walmsley, 2009, Mad Scientists bend the frame of biobank governance in British Columbia | Not CJ |
